# Sequence Diversity in the *Dickeya fliC* Gene: Phylogeny of the *Dickeya* Genus and TaqMan® PCR for *'D. solani'*, New Biovar 3 Variant on Potato in Europe

**DOI:** 10.1371/journal.pone.0035738

**Published:** 2012-05-03

**Authors:** Johan Van Vaerenbergh, Steve Baeyen, Paul De Vos, Martine Maes

**Affiliations:** 1 Unit Plant Sciences-Crop Protection, Institute for Agricultural and Fisheries Research-ILVO, Merelbeke, Belgium; 2 Laboratory of Microbiology, Ghent University, Ghent, Belgium; 3 BCCM/LMG Bacteria Collection, Laboratory of Microbiology Ghent University, Ghent, Belgium; Kansas State University, United States of America

## Abstract

Worldwide, *Dickeya* (formerly *Erwinia chrysanthemi*) is causing soft rot diseases on a large diversity of crops and ornamental plants. Strains affecting potato are mainly found in *D. dadantii*, *D. dianthicola* and *D. zeae*, which appear to have a marked geographical distribution. Furthermore, a few *Dickeya* isolates from potato are attributed to *D. chrysanthemi* and *D. dieffenbachiae*. In Europe, isolates of *Erwinia chrysanthemi* biovar 1 and biovar 7 from potato are now classified in *D. dianthicola*. However, in the past few years, a new *Dickeya* biovar 3 variant, tentatively named ‘*Dickeya solani*’, has emerged as a common major threat, in particular in seed potatoes. Sequences of a *fli*C gene fragment were used to generate a phylogeny of *Dickeya* reference strains from culture collections and with this reference backbone, to classify pectinolytic isolates, i.e. *Dickeya* spp. from potato and ornamental plants. The reference strains of the currently recognized *Dickeya* species and ‘*D. solani*’ were unambiguously delineated in the *fli*C phylogram. *D. dadantii*, *D*. *dianthicola* and ‘*D. solani*’ displayed unbranched clades, while *D. chrysanthemi*, *D. zeae* and *D. dieffenbachiae* branched into subclades and lineages. Moreover, *Dickeya* isolates from diagnostic samples, in particular biovar 3 isolates from greenhouse ornamentals, formed several new lineages. Most of these isolates were positioned between the clade of ‘*D. solani*’ and *D. dadantii* as transition variants. New lineages also appeared in *D. dieffenbachiae* and in *D. zeae*. The strains and isolates of *D. dianthicola* and ‘*D. solani*’ were differentiated by a *fli*C sequence useful for barcode identification. A *fli*C TaqMan®real-time PCR was developed for ‘*D. solani*’ and the assay was provisionally evaluated in direct analysis of diagnostic potato samples. This molecular tool can support the efforts to control this particular phytopathogen in seed potato certification.

## Introduction

The genus *Dickeya* was established by the reclassification of *Pectobacterium* (*Erwinia*) *chrysanthemi* and *Brenneria paradisiaca* as *D. chrysanthemi* and *D. paradisiaca*, respectively and for the accommodation of four new species, i.e. *D. dadantii*, *D. dianthicola*, *D. dieffenbachiae* and *D. zeae*; based on analysis of 16S rRNA gene sequences, DNA:DNA reassociation kinetics and phenotypic features including biochemical and serological reactions [1]. Multi Locus Sequence Analysis underpinned that *Dickeya* constitutes a distinct genetic clade in the soft rot *Enterobacteriaceae* [2]. *Dickeya* species are broad host range phytopathogens which principal disease symptom is maceration of plant tissues due to pectinolytic activity [2], [3]. Strains affecting potato are mainly found in three *Dickeya* species, i.e. *D. dadantii* (biovar 3), *D. dianthicola* (biovars 1 and 7) and *D. zeae* (biovar 3). A few strains are assigned to *D. chrysanthemi* (biovar 5 and 6) and to *D. dieffenbachiae* (biovar 2) [4]. *Erwinia chrysanthemi* is known in potato production in some European countries for over 40 years and is associated with slow wilt and internal stem necrosis. These strains are now assigned to *D. dianthicola* [3], [5]. However, in the past few years a new *Dickeya* biovar 3 strain, tentatively named ‘*Dickeya solani*’, has emerged as a common major threat, in particular on seed potatoes [5]. Across wide environmental conditions it causes extensive maceration of the seed tuber, rapid wilting and blackleg-like symptoms in the stem with decomposition of the pith. Both *D. dianthicola* and ‘*D. solani’* are disseminated by infected or contaminated seed tubers. Seed certification generally implements a zero tolerance for blackleg in field inspections of high grade seed.

A phylogenetic analysis using concatenated *atp*D, *car*A and *rec*A loci resolved the relatedness in the plant pathogenic *Enterobacteriaceae* [6]. Although *Dickeya* formed a contiguous clade with *Pectobacterium*, *Brenneria* and *Samsonia*, the gene amplicon sequences were sufficiently distinct to support the generic status of the taxon. Diagnostic identification of bacterial isolates as *Dickeya* is principally done by testing the production of indigoidin on specific culture medium [7], maceration of potato tuber tissue and the production of the 420 bp amplicon in a PCR based on the *pel*ADE gene cluster [8]. However, robust tools for the differentiation of *Dickeya* species were not available until fairly recently. In the past few years, several gene loci have been found to reliably differentiate species in several taxa of plant pathogenic bacteria, e.g. *Ralstonia solanacearum* [9], *Pseudomonas syringae* [10] and *Xanthomonas* [11]. The *rec*A locus was used for the first phylogenetic analysis of all species within the genus *Dickeya*, extending previous studies based on 16S rDNA [3]. It displayed new genomic clades and, in particular, a clonal delineation of an emerging biovar 3 variant isolated from potato in the past decade in Europe [12]. This new genetic clade was later on validated in a polyphasic analysis using *dna*X sequence data and genomic fingerprinting [5]. These studies suggest that this new variant may represent a new species for which the name ‘*Dickeya solani*’ is provisionally used, but it is not formally accepted yet [4]. Further evidence for the taxonomic discrimination of this separate biological unit may be derived from sequence information of genes involved in pathogenesis and virulence.

Many phytopathogenic bacteria are motile by means of flagella and flagellar genes contribute to virulence [13], [14], [15] and to host-pathogen interactions, i.e. for pectinolytic *Enterobacteriaceae* [16], [17], [18]. The flagellar filament is composed of a single protein, flagellin, which is encoded by the *fli*C gene. The flagellin proteins contribute to antigenic variation [19] that is also displayed in *Dickeya* [20], [21], [22]. More than 10 different serogroups have been identified [23] and differences were found among *Dickeya* isolates from potato [24]. Sequence variability of the *fli*C gene has been used to differentiate among isolates of several clinical bacterial species [25], [26], [27] and the bacterial phytopathogen *R. solanacearum* [28], for molecular typing and phylogenetic analysis [29], for taxonomic applications [30] and it showed potential as a biomarker for phylogenetic and epidemiological studies [31].

This paper reports on the application of *fli*C sequences to differentiate *Dickeya* strains at the species and infraspecific level and to specifically diagnose ‘*D. solani*’ with a *fli*C barcode or TaqMan® real-time PCR.

## Results

### FliC phylogeny of the reference strains

**Figure 1:**
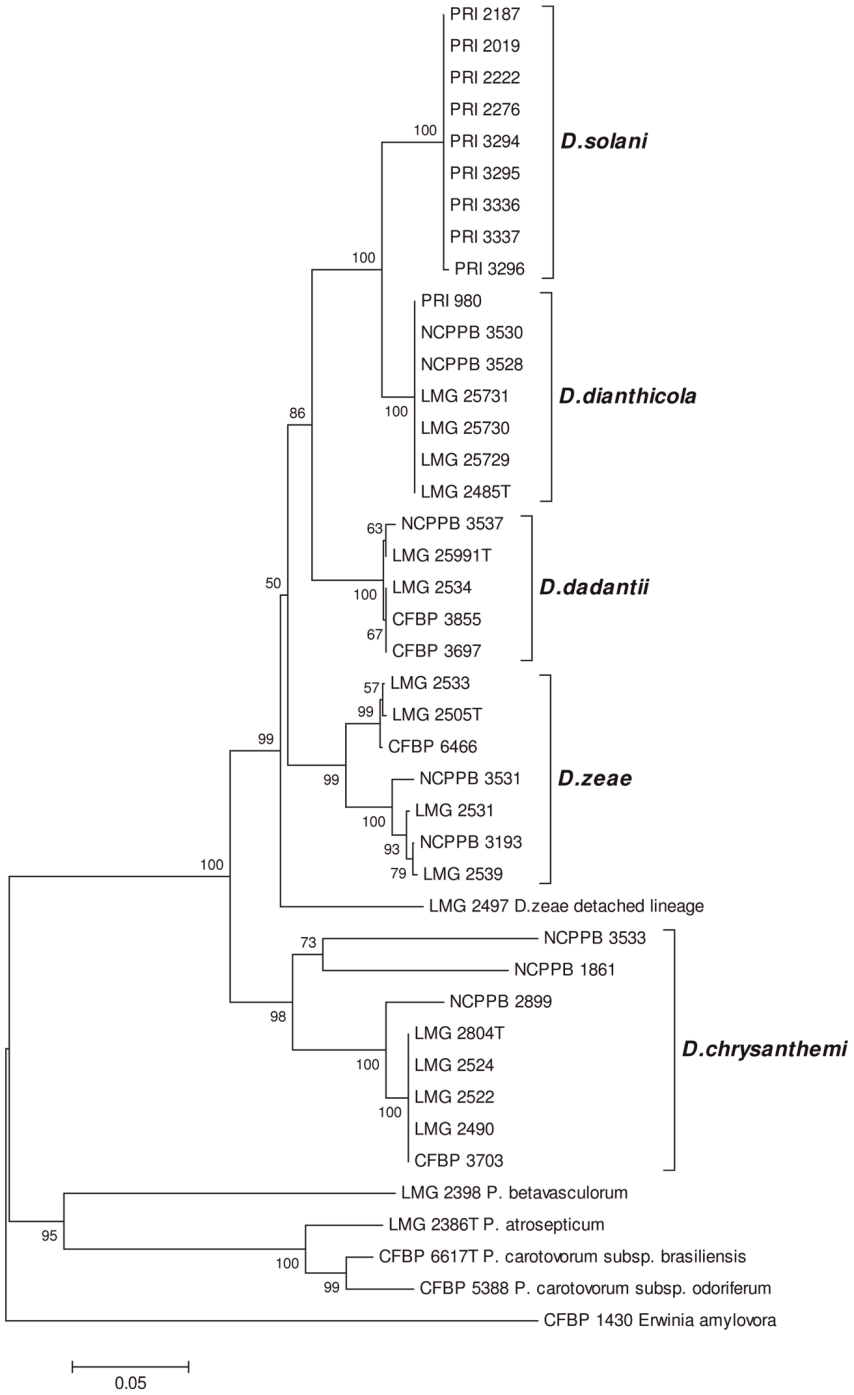
Conventional phylogenetic analysis produced by the neighbour-joining method of *fli*C amplicon sequences with *fli*C-1/*fli*C-2 primers [**37**] for reference strains of the recognized *Dickeya* taxa, except *D. dieffenbachiae* (*D. dadantii* subsp. *dieffenbachiae*) and *D. paradisiaca*, of ‘*D. solani*’, and taxa of *Pectobacterium*. Bootstrap values after 1000 replicates are expressed as percentages. The scale bar indicates the fraction of substitutions per site.

The *fli*C-1/*fli*C-2 primers were used to produce the reference PCR amplicon of the *fli*C gene. The results for the individual strains are presented in Table S1. A single amplicon of approximately 650 bp was produced for all strains tested of *D*. *chrysanthemi*, *D*. *dadantii*, *D*. *dianthicola*, ‘*D. solani*’ and *D*. *zeae*. The amplicon sequences correspond to the *fli*C ORF region of *D. dadantii* 3937 strain (GenBank accession CP002038.1). A single amplicon of approximately 900 bp was obtained for the strains of *D*. *paradisiaca*. It shows homology with flagellin gene sequences of *Dickeya* strain Ech703 (complete genome sequence in GenBank accession CP001654). It did not reveal, however, a significant homology with *fli*C sequences in other available *Dickeya* genomes, i.e. *D. dadantii* 3937, *Dickeya* strain Ech586 and *Dickeya* strain Ech1591. *D. paradisiaca* has a limited biological and geographical distribution. Apparently strains have not been isolated over the past 30 years. Furthermore, the strains tested did not exhibit indigoidin production on NGM nor a genuine maceration activity in potato. Multiple amplicons were produced for the strains of *D*. *dieffenbachiae*. These did not share a valid homology with the *fli*C sequence of *D. dadantii* 3937. Ultimately, global sequence alignment was performed with 621 bp consensus sequences for all *Dickeya* reference strains, except for those of *D. dieffenbachiae* and *D. paradisiaca*. The customised phylogenetic relatedness is displayed in Figure 1. A separate clade with a clonal structure and a single sequevar is formed by the strains of ‘*D. solani*’, which are isolates from potato in Europe and Israel and one isolate from hyacinth in The Netherlands. The *D. dianthicola* reference strains also form a single clade with a single sequevar, regardless their different biological and geographical origin. *D. dianthicola* shows a very close relationship with ‘*D. solani*’. Both are most related to the strains of *D. dadantii which form a* third clade. Although biologically and geographically quite diverse, *D. dadantii* strains also constituted a single sequevar. The *D. zeae* reference strains are attributed to two sub-clades and to a separate branch. The first sub-clade, phylotype 1 (P1), represents a single sequevar and contains strains isolated on the American and European continent. The second sub-clade, phylotype 2 (P2), also represents a single sequevar and consists of strains isolated on the Asian and Australian continent. Strain LMG 2497 isolated from sweet corn in the USA is attributed to a detached lineage of *D. zeae* and is considered a separate sequevar. The *D. chrysanthemi* reference strains form an aggregate clade with the type strain, other strains from chrysanthemum and strains from euphorbia, sunflower and carrot in a large sub-clade containing two sequevars. A biovar 6 strain from *Parthenium* and a strain from potato, both isolated in the USA, form a dichotomous branch in the *D. chrysanthemi* clade. An aggregate clade of *Pectobacterium* was formed containing *P. betavasculorum*, *P. atrosepticum*, *P. carotovorum* ssp. *odoriferum* and *P. carotovorum* ssp. *brasiliensis*. A *fli*C amplicon was not produced for the type strain of *Pectobacterium carotovorum* ssp. *carotovorum* and *Pectobacterium wasabiae*, nor for the potato associated bacteria tested.

### FliC-based identification of Dickeya isolates

**Figure 2:**
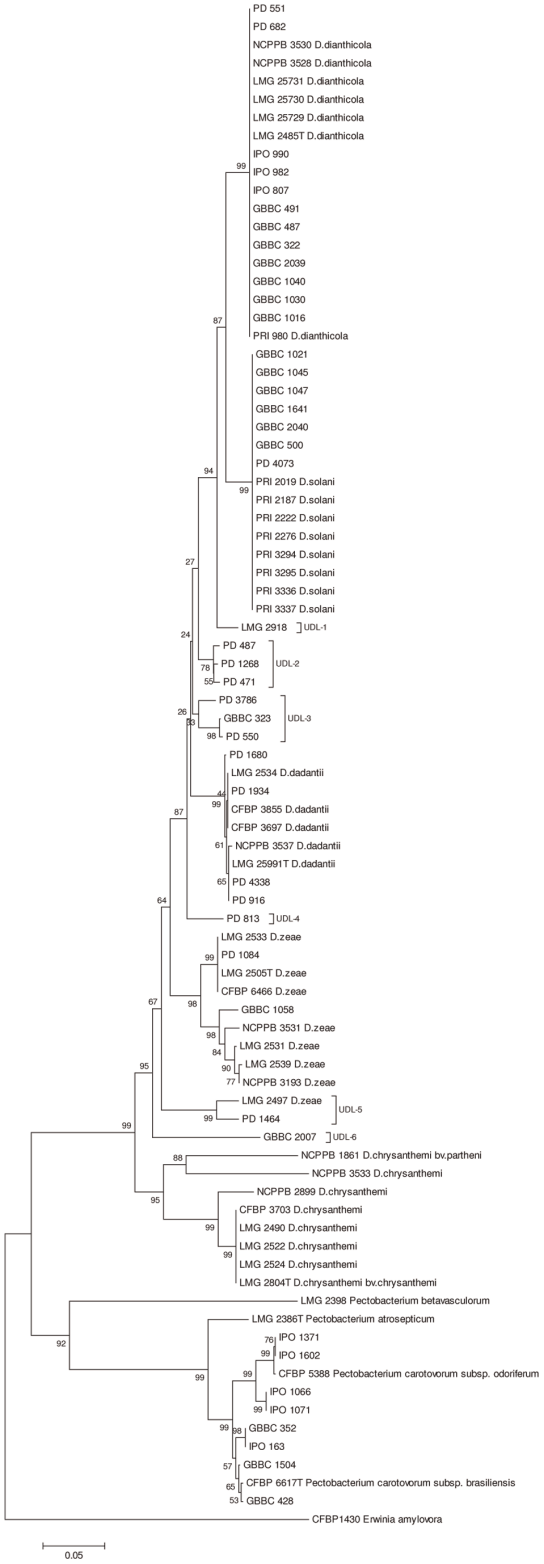
Conventional phylogenetic analysis produced by the neighbour-joining method of *fli*C amplicon sequences with *fli*C-1/*fli*C-2 primers [**37**] for reference strains of the recognized *Dickeya* taxa, except *D. dieffenbachiae* (*D. dadantii* subsp. *dieffenbachiae*) and *D. paradisiaca*, of ‘*D. solani*’ and *Dickeya* and *Pectobacterium* isolates from diagnostic samples. Bootstrap values after 1000 replicates are expressed as percentages. The scale bar indicates the fraction of substitutions per site.

From the fifty isolates obtained from diagnostic samples, thirty-eight were attributed to *Dickeya* and twelve to *Pectobacterium* on the basis of indigoidin production on NGM, maceration of potato tuber tissue and a PCR amplicon produced with *pe*lADE or *pel*Y primers respectively. Subsequently, *fli*C amplicons were obtained and sequenced. The *fli*C phylogeny of the reference strains was used as backbone to position the isolates. All isolates preliminary identified as *Dickeya* with the above mentioned methods, were validated by their position in the phylogenetic *fliC* tree. The results are displayed in Figure 2 and in Table S2. The *Dickeya* isolates from potato were either classified in the *D. dianthicola* clade or in the ‘*D. solani*’ clade. Furthermore, the *fli*C sequences were identical for all strains tested of *D. dianthicola* and ‘*D. solani*’. The consensus sequences exhibit twenty-five different signature positions which provide reliable barcodes to allocate isolates to one of these clades. *Dickeya* strain LMG 2918, isolated from *Phalaenopsis* orchids, is attributed to a separate branch, which is considered as an unassigned *Dickeya* lineage (UDL-1). Thirteen *Dickeya* biovar 3 isolates from greenhouse ornamentals exhibited substantial sequence variation. Six of those isolates are classified in two additional unassigned *Dickeya* lineages (UDL-2 and UDL-3). Four isolates are classified in the *D. dadantii* clade and one isolate is assigned to the *D. zeae* phylotype 1 sub-clade. Furthermore, another *Dickeya* biovar 3 isolate from *Phalaenopsis* orchids constitutes a fourth unassigned lineage (UDL-4) and one from Freesia clusters in the separate lineage with strain LMG 2497 from sweet corn which is now specified as UDL-5. A *Dickeya* biovar 3 isolate from corn in Belgium is assigned to the *D. zeae* phylotype 2 sub-clade and, finally, a *Dickeya* biovar 3 isolate from Belgian lettuce is placed in yet another unassigned lineage (UDL-6). These fifteen *Dickeya* biovar 3 isolates represent seven additional sequevars. The eighteen *Dickeya fli*C sequevars determined in this study are listed with their associated GenBank accession numbers in Table S3. Eight *Pectobacterium* isolates are assigned to the aggregate cluster containing *P. carotovorum* ssp. *odoriferum* and *P. carotovorum* ssp. *brasiliensis*. The *fli*C amplicon was not produced for four *Pectobacterium* isolates from potato.

### FliC-based identification of Dickeya dieffenbachiae

**Figure 3:**
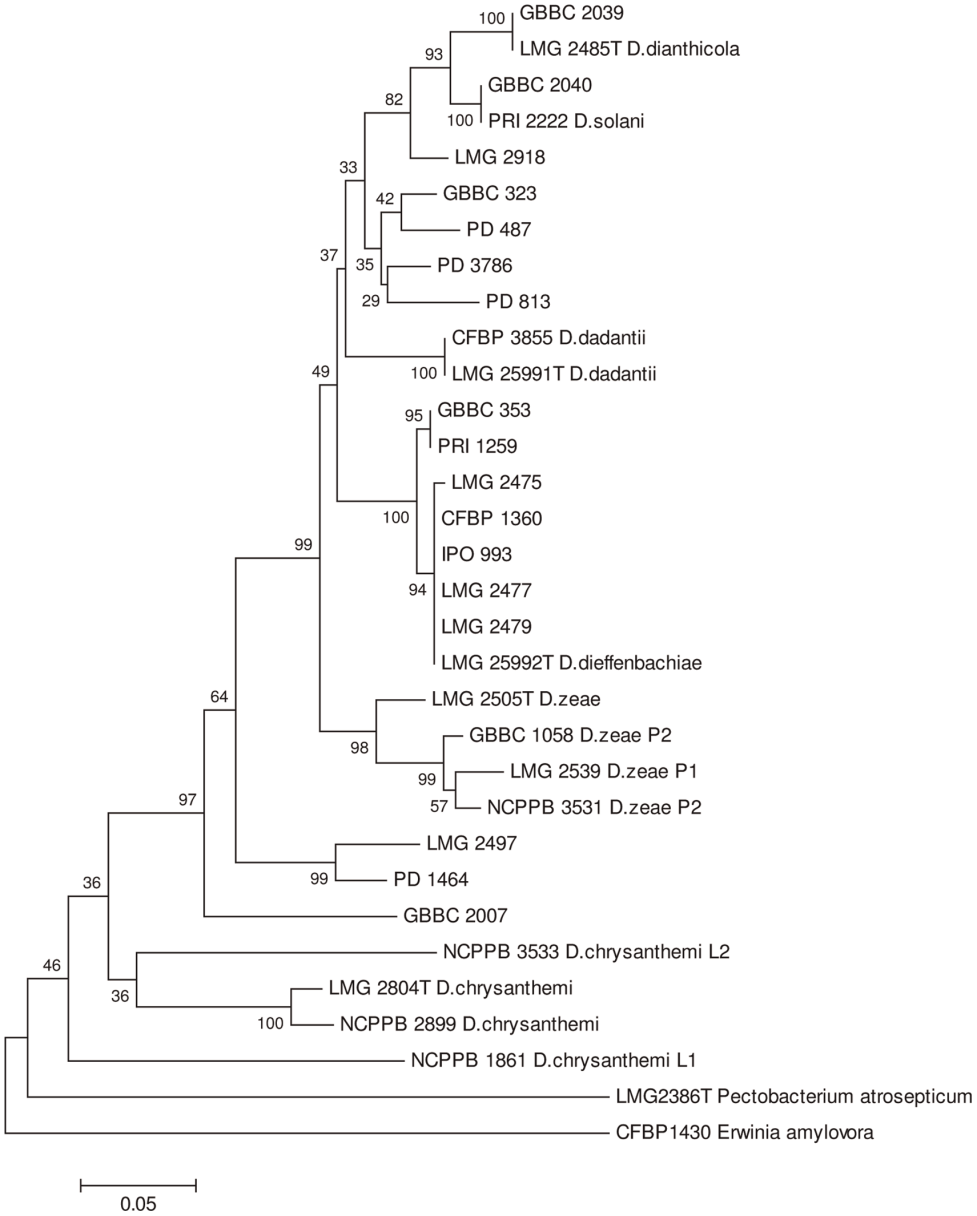
Conventional phylogenetic analysis produced by the neighbour-joining method of *fli*C amplicon sequences with fliC-for/fliC-rev primers [**38**] for reference strains and isolates of *Dickeya dieffenbachiae* (*D. dadantii* subsp. *dieffenbachiae*) and positioning of the *Dickeya* sequevars determined with the fliC-1/fliC-2 primers [**37**]. Bootstrap values after 1000 replicates are expressed as percentages. The scale bar indicates the fraction of substitutions per site.

A second primer set (*fli*C-for/*fli*C-rev) was used for *D. dieffenbachiae* to produce a single *fli*C amplicon (∼370 bp) located inside the ∼650 bp *fli*C amplicon. Comparative sequence analysis was done on 353 bp consensus sequences which were used to position the *D. dieffenbachiae* strains and isolates in the background of the eighteen *Dickeya* sequevars identified for the reference *fli*C fragment. The phylogram of *fli*C sequences trimmed at the shorter fragment is displayed in Figure 3. *D. dieffenbachiae* displayed an aggregate clade with two sub-clades. One contains the strains isolated from *Dieffenbachia* and a Dutch isolate from potato and exhibits an almost clonal structure. Another isolate from potato was attributed to a second sub-clade together with a Belgian isolate from *Dieffenbachia* sp. The *D. dieffenbachiae* clade showed a high degree of relatedness to *D. dadantii*.

### FliC TaqMan® PCR for identification of ‘Dickeya solani’

The TaqMan® real-time PCR for ‘*D. solani*’ was designed to amplify a 112 bp stretch of the *fli*C amplicon (Table 1). The assay was applied on the fifty–six reference strains and the fifty diagnostic isolates. Positive reactions, attested by C_t_-values ≤25, were only obtained for the nine reference strains and the seven diagnostic isolates of ‘*D. solani*’. Negative results, demonstrated by the absence of a C_t_ -value after 40 PCR cycles, were obtained for all other bacterial cultures tested. The qualitative results of the *fli*C TaqMan® PCR are given in Table S1 and S2.

**Table 1:** Primers for conventional *fli*C PCR and primers and TaqMan probe for identification and diagnosis of ‘*D. solani*’.

primer or probe	sequence (5′ 3′)	reference	derived from	amplicon	use
*fli*C-1	TATCAACAGCGCCAAAGACAACGC	37	*D. dadantii* CFBP 3855	∼650 bp	PCR & sequencing
*fli*C-2	ACGGCTCATGTTGGATACTTCGTT				
*fli*C-for	GACCGTACTGCAATCCAGC	38	*D. dadantii* CFBP 3855	∼370 bp	PCR & sequencing
*fli*C-rev	CTGGAAGCGGTTCAGAGT				
ds-f	GCGAACTTCAACGGTAAA	this study	‘*D. solani*’ GBBC 2040	112 bp	TaqMan® real-time PCR
ds-r	CAGAGCTACCAACAGAGA				
ds-p*	CTCTGCTGGACGGTTC				

* probe.

### Fast fliC TaqMan PCR diagnosis of ‘Dickeya solani’ in symptomatic potato tissue

Thirty diagnostic samples from seed potato production in Flanders were analysed in this study. They consisted of either wilting potato stems with blackleg symptoms, necrosis and maceration of the stem pith or wilting stems without blackleg but with a macerated mother tuber. A fast diagnostic procedure for ‘*Dickeya solani*’ was performed by applying the *fli*C TaqMan® PCR without prior enrichment of the sample extracts. The test results were compared with the conventional diagnostic protocol in which the bacteria were cultured from the sample extract by dilution plating and further characterization of the isolates in phenotypic and molecular tests. The results are summarized in Table 2. Definite positive results in the *fli*C TaqMan® PCR, attested by 18.1< C_t_ <28.1, were obtained for twelve out of the thirty sample extracts, diagnosing the presence of ‘*D. solani*’. In the conventional diagnostic protocol, ‘*D. solani*’ was indeed isolated as a distinct colony morphotype from these samples and sequence analysis of the *fli*C amplicon produced with primers *fli*C-1/*fli*C-2 confirmed the identity. The ‘*D. solani*’ morphotype was not isolated from the other eighteen sample extracts, which underpinned the negative reactions in the *fli*C TaqMan® PCR on the extracts. Furthermore, seven of these samples were found to contain a different *Dickeya* variant producing indigoidin on NGM medium, maceration of potato tuber tissue and the *pel*ADE amplicon in PCR. These isolates were identified as *D. dianthicola* by sequence analysis of the *fli*C amplicon. *Pectobacterium* spp. was diagnosed in the remaining eleven samples, as confirmed by maceration of potato tuber tissue, the absence of indigoidin production on NGM medium, a positive reaction in the *pel*Y PCR and a negative reaction in the *pel*ADE PCR. More detail of the diagnostic tests are presented in Table S4.

**Table 2:** Analysis of diagnostic samples of seed potato plants showing wilting, blackleg or tuber maceration symptoms.

# samples	morphotype on PDA	potato maceration	indigoidin op NGM	*pel*ADE	*pel*Y	*fli*C qPCR extract	*fli*C qPCR culture	result
7	A	+	+	+	−	−	−	*D. dianthicola* 1
12	B	+	+	+	−	+	+	‘*D. solani*’
11	C/D	+	−	−	+	−	−	*Pectobacterium* sp.

1 The isolates of D. dianthicola were identified by sequencing of the fliC PCR amplicon.

## Discussion

In less than a few years, a biovar 3 of *Dickeya*, provisionally named ‘*Dickeya solani*’, has become the predominate cause of wilting, blackleg and tuber maceration of potato in several European countries. The new form has been described as more aggressive, more likely to infect at lower cell densities and to spread from plant to plant along and even across plant rows, and causing damage in a wider range of conditions than observed for the ‘traditional’ blackleg (*Pectobacterium atrosepticum*) or for *Dickeya dianthicola* which is known for over 40 years in potato in some European countries [4]. Consequently, the blackleg tolerance in crops of basic and certified seed potatoes has been substantially reduced. Some countries even implement protective measures to prevent the introduction of ‘*D. solani*’ with seed potatoes imported from countries where the pathogen is known to occur. Diagnostic tests should provide rapid and reliable identification of ‘*D. solani*’ and should discriminate it from *Pectobacterium* and other *Dickeya* taxa, mainly *D. dianthicola*, which can cause blackleg and potato tuber maceration as well. This paper provides a method that is based on the *fli*C gene which codes for the flagellar subunit protein flagellin. The *fli*C gene can exhibit considerable intra-species sequence variation that can be used for identification at an infra-species or even strain level [31]. Phylogenetic analysis of the *fli*C amplicons unambiguously differentiated five of the six currently recognized *Dickeya* species [1] and ‘*D. solani*’, with most branches supported by high bootstrap values. In this respect, *fli*C sequence comparison confirmed that ‘*D. solani*’ is clearly a new, separate and clonal clade within the genus. The short phylogenetic branches indicate the relatively close relatedness of *D. dianthicola*, *D. dadantii* and ‘*D. solani*’, suggesting that taxonomically these taxa may well be delineated at subspecies level. The *fli*C phylogeny does not extensively support that ‘*D. solani*’ deserves a separate species status according to the current bacterial species concept [32]. Larger *fli*C sequence variation exist within *D. zeae* with two phylotypes and within *D. chrysanthemi as* demonstrated in the *rec*A phylogeny [3]. A strain isolated from sweet corn is assigned to a lineage detached of *D. zeae*. This strain was classified as *D. zeae* phylotype 1 in the *rec*A phylogram [3]. All other corn strains of *D. zeae* in the *fli*C clade were isolated from maize varieties used for livestock fodder or processing and thus the contrasting position in the *fli*C phylogram may reflect the association of *fli*C to host designation. The *D. chrysanthemi* strains clustered in an aggregate clade with a large sub-clade identified as *D. chrysanthemi* biovar *chrysanthemi* and a detached *D. chrysanthemi* biovar *parthenii* lineage according to the species description [1]. The *D. chrysanthemi* strain isolated from potato in the USA is more related to the ‘*parthenii*’ biovar than to the ‘*chrysanthemi*’ biovar. The assignment of *D. dieffenbachiae* in the *fli*C phylogeny was performed with an amplicon internal to the regular 650 bp amplicon. The grouping of *Dickeya* taxa and sequevars remained stable. The strains and isolates of *D. dieffenbachiae* are closely related to *D. dadantii* which underpins the recent re-classification of *D. dieffenbachiae* as *D. dadantii* ssp. *dieffenbachiae* [33]. The robustness of the *fli*C phylogeny is further underpinned by analogies in phylogenies based on *rec*A [3] and *dna*X [5] loci, presented in Table S5.

The isolates from diagnostic samples were introduced the *fli*C phylogram allowing their assignment to a *Dickeya* taxon. All isolates from potato were assigned to *D. dianthicola* or ‘*D. solani*’ and accentuated the clonal structure of these two clades. *Dickeya* biovar 3 isolates from various ornamentals exhibited significant sequence variability which resulted in six unassigned lineages and, typically, strains isolated from the same plant family (the *Araceae*, with *Philodendron* and *Aglaonema*) constituted one of these lineages (UDL-2). So, again not totally unexpected, *fli*C-based bacterial typing could be informative on different plant hosts of strains. Furthermore, three of these lineages are positioned in between ‘*D. solani*’ and *D. dadantii*, the latter being biologically and geographically the most diverse *Dickeya* taxon [3]. Although the phylogenetic significance of these up to now not formally classified lineages remains to be clarified, it is our opinion that the presented data allude to the origin of ‘*D. solani*’ as being from one of the variants existing on ornamentals which then spread clonally in potato. Alternatively, *fli*C sequence drift when residing on different plant hosts could be responsible for the existence of the unassigned lineages. The apparent pectinolytic activity of *Dickeya* spp. make them broad host range pathogens which increases the potential for genetic exchange as a result of adaptation to a different environment, i.e. a new plant host [34]. Furthermore, the short branch lengths in the *fli*C phylogeny tend to reveal that *D. dianthicola*, ‘*D. solani*’, *D. dadantii* and the unassigned lineages UDL-1, UDL-2, UDL-3 and UDL-4 are a species complex. Further analysis should clarify the taxonomic position of these taxa, i.e. the classification at subspecies level as already done for the *D. dadantii* – *D. dieffenbachiae* aggregate [33].

The apparent clonal structure of the ‘*D. solani*’ clade enabled the development of a TaqMan® PCR to specifically identify this variant and for its direct diagnosis in symptomatic potato stems and tubers. The primers are positioned in the more variable stretches of the *fli*C gene amplicon and the TaqMan® probe is situated in a region with two single nucleotide polymorphisms. Furthermore, the 3′ MGB probe is more appropriate for single base mismatches, thus increasing the specificity of the assay. ‘*D. solani*’ is differentiated from all other reference strains and isolates, i.e. from *D. dianthicola* isolates from potato and from the *Dickeya* biovar 3 isolates from greenhouse ornamentals which were attributed to unassigned lineages. However, it did not differentiate the ‘*D. solani*’ isolate from a hyacinthus bulb which sets focus on the relation of this variant with the cultivation of bulb-producing ornamentals. The specificity of the molecular assay for ‘*D. solani*’ was also demonstrated in direct analysis of potato samples. Such diagnostics are essential if legislation that imposes a zero tolerance in seed potatoes is to be effective. The assay has not yet been validated for detection of latent infections and is, pending the outcome of these tests, proposed here as a diagnostic tool.

### Conclusions

The sequence diversity of the *Dickeya fli*C gene produced a phylogeny of the currently recognized *Dickeya* taxa and the new *Dickeya* biovar 3 variant from potato in Europe, tentatively named ‘*D. solani’*. *Dickeya* isolates from diagnostic samples were introduced into this phylogenetic backbone displaying new, unassigned lineages in the *fli*C phylogeny, in particular of certain *Dickey*a biovar 3 isolates from ornamentals which were positioned as ‘*D. solani*’ – *D. dadantii* transition variants. These may have spread into potato and become clonally established as ‘*D. solani*’ by seed potato propagation. A TaqMan® real-time PCR was developed on the unique *fli*C sequence of ‘D.solani’ and provisionally evaluated. This diagnostic tool was effective for diagnosis of ‘*D. solani*’ in potato plants and tubers.

## Materials and Methods

### Bacterial strains

The strains and isolates used are listed in Tables S1 and S2. The reference set (Tables S1) consisted of strains from the six currently recognized *Dickeya* species, typed *Dickeya* isolates from the new clade of biovar 3 strains from potato (‘*D. solani*’), strains from the *Pectobacterium* taxa and strains of *Clavibacter michiganensis* subsp. *sepedonicus*, *Ralstonia solanacearum* and *Paenibacillus macerans*. Most reference strains were acquired from public and certified culture collections, while the isolates of ‘*D. solani*’ were obtained in the framework of the European *Dickeya* consortium (*Dickeya* Research Network hosted by the James Hutton Institute, Dundee, Scotland, UK). The second set (Table S2) consisted of *Dickeya* and *Pectobacterium* isolates from diagnostic samples that were obtained from the Diagnostic Centre for Plants of ILVO (GBBC numbers) and from diagnostic culture collections in The Netherlands (PD and IPO/PRI numbers). These isolates were mainly recovered from symptomatic potato plants and tubers and from greenhouse ornamentals. All strains and isolates were archived in cryopreservation.

The reference strains were first cultured on Difco LB agar (Miller's modification) and then subcultured on nutrient sucrose agar (NSA  =  Difco Nutrient Agar supplemented with 5% sucrose). *Dickeya* strains were verified with the *Dickeya* genus specific *pel*ADE primers [8], used in colony PCR. A single colony from a 48 hr culture on NSA was suspended in 1 ml of sterile 10 mM phosphate buffer (PB) pH 7.2 and DNA was obtained by alkaline lysis [35]. After pulse centrifugation to sediment cell debris, two microliters of the supernatant were used in the PCR reactions.

The isolates from diagnostic samples were cultured on nutrient glycerol agar supplemented with manganese chloride (NGM) for production of indigoidin pigment, which is characteristic for *Dickeya* spp. [7]. The macerating properties of the isolates were determined on potato tubers ‘Spunta’ derived from minitubers. Cell suspensions with density of approximately 10^7^ colony forming units per ml were prepared in sterile 10 mM PB. A conical tissue core was removed at the heel end of the tubers and 100 µl of the cell suspension was pipetted onto the cut surface. The tissue cone was reinstalled after the applied volume was absorbed and the cone was then tightened by parafilm tape. Three tubers were used for each isolate in one unreplicated assay. They were placed in moist sterilised white sand in an appropriate receptacle that was closed with a lid and aerobically incubated for 48 hours at 28°C. Macerative isolates producing the indigoidin pigment were further identified as *Dickeya* spp. with the *pel*ADE PCR. Macerative isolates not producing the indigoidin pigment were further tested with the *pel*Y PCR to identify *Pectobacterium* strains [36]. PCRs were performed on bacterial DNA prepared as described above for the collection strains.

### Conventional fliC PCR and amplicon sequencing

Single bacterial colonies were transferred in 3 ml LB broth and grown in a shaking incubator (200 rpm) at 28°C. DNA was isolated from overnight broth cultures using the Qiagen DNeasy Blood & Tissue kit as described by the manufacturer, including the pre-treatment for gram-negative bacteria. DNA concentration and quality (according to A_260/280_ and A_260/230_ ratios) were assessed using a Nanodrop ND-1000 spectrophotometer. Isolated DNA was adjusted to approximately 50 ng/µl. The *fli*C gene fragment was amplified with PCR primers (Table 1) designed for the *Dickeya dadantii* 3937 strain. PCR with the *fli*C-1 and *fli*C-2 primers [37] was performed with 5 µl DNA template in 1 x Faststart High Fidelity reaction buffer (Roche Applied Science) with 2 mM MgCl_2_, 0.2 mM of each dNTP, 0.2 µM of each primer, 1 unit of FastStart Taq DNA polymerase (Roche Applied Science) and sterile molecular-grade water for a total volume of 50 µl. PCR was performed in a Bio-Rad Laboratories C1000 thermal cycler with initial denaturation at 95°C for 4 minutes, followed by 35 cycles of 95°C for 30 seconds, 55°C for 1 minute and 72°C for 45 seconds, and a terminal extension step of 7 minutes at 72°C and subsequent cooling to 12°C. PCR with the *fli*C-for and *fli*C-rev primers [38] was performed with 5 µl DNA template in 1 x OneTaq standard reaction buffer (New England Biolabs) with 2 mM MgCl2, 0.2 mM of each dNTP, 0.2 µM of each primer, 1,25 unit of OneTaq Hotstart polymerase (New England Biolabs) and sterile molecular-grade water for a total volume of 50 µl. PCR was performed in a Bio-Rad Laboratories C1000 thermal cycler with initial denaturation at 94°C for 30 seconds, followed by 30 cycles of 94°C for 30 seconds, 53°C for 1 minute and 68°C for 30 seconds, and a terminal extension step of 5 minutes at 68°C and subsequent cooling to 12°C.

PCR amplicons were resolved by electrophoresis in a 1.5% agarose gel stained with ethidium bromide. PCR amplicons were extracted from gel with the Nucleospin Extract II kit (Macherey-Nägel). DNA concentration and quality were assessed in a Nanodrop ND-1000 spectrophotometer. Purified PCR amplicons were sequenced in both directions by a commercial sequencing service (Macrogen Ltd, Korea), using the same primer set as for PCR amplification.

### Sequence alignment and phylogenetic analysis

The *fli*C consensus sequences were delineated by clipping the PCR amplicon sequences to a standard start position [A(A/G)TC(A/G)GC(A/G)T at 5′ end] and finish position [TG(A/G/C)G(C/A) (A/G)G(T/A)(C/T)AT(A/G) at 3′ end]. Phylogenetic and molecular evolutionary analysis were conducted using MEGA version 5 software [39]. Sequence alignment of the trimmed sequences was done using the clustalW algorithm [40] in MEGA 5 and phylogenetic trees were generated using the neighbour-joining, maximum parsimony and maximum likelihood algorithm [41]. Distance estimation was calculated using the p-distance substitution model [42] with 1000 bootstrapping replications. Based on the sequence distances, *fli*C clades were differentiated by monophyletic clustering [43] with type strains and reference strains. Sequevars were designated within these clades on the basis of at least 1% sequence difference [9]. Sequevar sequences were submitted to GenBank (Table S3). The *fli*C sequence of *Erwinia amylovora* CFBP 1430 (GenBank accession AY743588) was used to root the phylogenies.

### FliC TaqMan® real-time PCR for ‘Dickeya solani’

Primers and TaqMan® MGB (5′-FAM/3′-BHQ1) probe (Life Technologies) specific for the *fli*C amplicon of ‘*D. solani*’ (Table 1) were designed with Premier Biosoft's Allele ID version 7 software. The real-time PCR was performed in a 25 µl volume in a MicroAmp Optical 96 well reaction plate with Optical Caps (Life Technologies). Briefly, 2 µl DNA template was added to 12,5 µl Taqman Gene Expression master mix 2x, 0,5 µl of primers Dsf and Dsr (15 µM), 0,5 µl of probe Dsp (10 µM) and molecular-grade water up to a final volume of 25 µl. Amplification and signal detection was done in an ABI Prism 7900HT Sequence Detection System (Life Technologies). The cycling profile is consisted of 2 minutes at 50°C for UNG-activation, 10 minutes at 95°C followed by 40 cycles of 15 seconds at 95°C and 1 minute at 63°C. The specificity of the TaqMan assay was tested by colony PCR with all reference strains and diagnostic isolates. Finally, suspensions of about 10^6^ colony forming units per ml in sterile 10 mM PB were tested as described for *pel*ADE and *pel*Y PCR using 2 µl of target per TaqMan PCR reaction.

### Molecular diagnosis of ‘Dickeya solani’ in symptomatic potato samples

Thirty diagnostic samples were analysed. For classical diagnosis, pathogen identification was done by isolation and further characterisation of the dominant bacterial type cultured upon plating of serial decimal dilutions of the extract from the symptomatic tissue. Therefore, minute quantities of affected tissue were aseptically removed at the margin of disease development in the stem or in the tuber and transferred in 1 ml of sterile 10 mM PB in a microvial. After vortexing of the preparation, dilution plating was performed on potato dextrose agar (Oxoid PDA) supplemented with cycloheximide. Isolated bacterial colonies representative at the higher extract dilutions were cultured on NGM and macerative properties were assessed as described before. Presumptive identification of macerative isolates was done by conventional *pel*ADE or *pel*Y PCR for *Dickeya* spp. or *Pectobacterium* spp. respectively. Suspensions of single colonies were prepared in 1 ml of sterile 10 mM PB and bacterial cells were subjected to alkaline lysis as explained above. *D. dianthicola* isolates were identified by sequencing of the PCR amplicon with the *fli*C-1 and *fli*C-2 primers and ‘*D. solani*’ isolates were identified in *fli*C TaqMan® real-time PCR as described.

The *fli*C TaqMan® real-time PCR was also performed directly on the potato sample extracts. The extract was allowed to settle for 15 minutes and then 100 µl was transferred in a 1.5 ml microvial and centrifuged for 10 minutes at 13000 g. The supernatant was removed and the pellet was resuspended in 100 µl 1 mM Tris-HCl pH = 8. DNA isolation was performed with the QuickPick™ Plant DNA kit (Bio-Nobile) using the Pickpen-8M magnetic tool according to the manufacterer's protocol for 100 mg starting material. The TaqMan PCR was performed using the protocol described above using 2 µl of eluted DNA as template.

## Supporting Information

Table S1 Reference strains of the recognized *Dickeya* taxa and ‘*D. solani*’, of *Pectobacterium* taxa and taxa of other phytopathogenic bacteria from potato, investigated in *fli*C phylogeny and *fli*C Taqman® real-time PCR. ^1^as identified in [1], [3] or [5] ^2^the strain in bold was used when different strain designations are displayed ^3^amplicon of the *fli*C locus with primers *fli*C-1 & *fli*C-2 [37]: ∼650 bp (+), multiple amplicons (+^a^), a larger amplicon of ∼900 bp (+^b^) or no amplicon (−) ^4^ *Dickeya* clades identified on the basis of global alignment of 621 bp *fli*C amplicon [37], except for *D.dieffenbachiae* which is identified on the basis of global alignment of 353 bp *fli*C amplicon [38] ^5^ *fli*C sequevar or sequence variant [9]: strains/isolates with >1% sequence variation in the 621 bp fragment. *D. dieffenbachiae* and *D. paradisiaca* are not considered in this classification ^6^negative result  =  no C_t_ LMG  =  Laboratory of Microbiology, Ghent University, Belgium NCPPB  =  National Collection of Plant Pathogenic Bacteria, York, UK CFBP  =  Collection Française des Bactéries Phytopathogènes, Angers France IPO/PRI  =  Plant Research International, Wageningen, The Netherlands
(XLS)

Table S2 Isolates of *Dickeya* and *Pectobacterium* from diagnostic samples investigated in *fli*C phylogeny and *fli*C Taqman® real-time PCR.^1^the strain in bold was used when different strain designations are displayed ^2^amplicon of the *fli*C locus with primers *fli*C-1 & *fli*C-2 [37]: ∼650 bp (+), multiple amplicons (+^a^), a larger amplicon of ∼900 bp (+^b^) or no amplicon (−) ^3^ *Dickeya* clades identified on the basis of global alignment of 621 bp *fli*C amplicon [37], except for *D. dieffenbachiae* which is identified on the basis of global alignment of 353 bp *fli*C amplicon [38] ^4^ *fli*C sequevar or sequence variant [9]: strains/isolates with >1% sequence variation in the 621 bp fragment. *D. dieffenbachiae* and *D. paradisiaca* are not considered in this classification ^5^negative result  =  no C_t_ GBBC  =  Culture collection of ILVO Diagnostic Centre for Plants (DCP) LMG  =  Laboratory of Microbiology, Ghent University, Belgium NCPPB  =  National Collection of Plant Pathogenic Bacteria, York, UK IPO  =  Plant Research International, Wageningen, The Netherlands
(XLS)

Table S3 *Dickeya fli*C sequevars and their associated Genbank accession numbers. UDL  =  Unassigned *Dickeya* Lineage.
(XLS)

Table S4 Diagnostic analysis of samples of seed potato plants showing wilting, blackleg or tuber maceration symptoms. ^1^Ct value ^2^The isolates attributed to *D. dianthicola* were identified by sequencing of the *fli*C amplicon [37].
(XLS)

Table S5 Agreement of *rec*A/*dna*X and *fli*C classification for 52 *Dickeya* strains and isolates. The *rec*A attribution was obtained from [11]. The *dna*X attribution was obtained from [5]. The *fli*C attribution is obtained in this study.
(XLS)
